# Early recurrence of atrial tachyarrhythmia after catheter ablation and its associations with clinical outcomes, atrial fibrillation burden, and blanking period duration: a *post hoc* analysis of the CABANA trial

**DOI:** 10.1093/europace/euag173

**Published:** 2026-07-07

**Authors:** Hongyu Liu, Runhui Hu, Wenhui Li, Eva Soler-Espejo, Tommaso Bucci, Yang Liu, Manlin Zhao, Tianshu Gu, Weiguo Fan, Bi Huang, Dhiraj Gupta, Douglas L Packer, Yang Chen, Gregory Y H Lip

**Affiliations:** Department of Cardiovascular Medicine, The Second Affiliated Hospital, School of Basic Sciences, Jiangxi Medical College, Nanchang University, Nanchang, Jiangxi, People’s Republic of China; Liverpool Centre for Cardiovascular Science at University of Liverpool, Liverpool John Moores University and Liverpool Heart & Chest Hospital, William Henry Duncan Building, 6 West Derby Street, Liverpool L7 8TX, UK; Department of Cardiovascular Medicine, The Second Affiliated Hospital, Jiangxi Medical College, Nanchang University, Nanchang, Jiangxi, People’s Republic of China; Liverpool Centre for Cardiovascular Science at University of Liverpool, Liverpool John Moores University and Liverpool Heart & Chest Hospital, William Henry Duncan Building, 6 West Derby Street, Liverpool L7 8TX, UK; Department of Computer Science, Faculty of Environment, Science and Economy, University of Exeter, Exeter, UK; Liverpool Centre for Cardiovascular Science at University of Liverpool, Liverpool John Moores University and Liverpool Heart & Chest Hospital, William Henry Duncan Building, 6 West Derby Street, Liverpool L7 8TX, UK; Department of Hematology, Hospital Clínico Universitario Virgen de la Arrixaca, University of Murcia, Instituto Murciano de Investigación Biosanitaria (IMIB-Arrixaca), Murcia, Spain; Liverpool Centre for Cardiovascular Science at University of Liverpool, Liverpool John Moores University and Liverpool Heart & Chest Hospital, William Henry Duncan Building, 6 West Derby Street, Liverpool L7 8TX, UK; Internal, Vascular and Emergency Medicine-Stroke Unit, University of Perugia, Perugia, Italy; Liverpool Centre for Cardiovascular Science at University of Liverpool, Liverpool John Moores University and Liverpool Heart & Chest Hospital, William Henry Duncan Building, 6 West Derby Street, Liverpool L7 8TX, UK; Department of Cardiovascular Medicine, The Second Affiliated Hospital, Jiangxi Medical College, Nanchang University, Nanchang, Jiangxi, People’s Republic of China; Jiangxi Provincial Key Laboratory of Molecular Medicine, the Second Affiliated Hospital, Nanchang, Jiangxi, People’s Republic of China; Liverpool Centre for Cardiovascular Science at University of Liverpool, Liverpool John Moores University and Liverpool Heart & Chest Hospital, William Henry Duncan Building, 6 West Derby Street, Liverpool L7 8TX, UK; Department of Cardiology, Beijing Anzhen Hospital, Capital Medical University, Engineering Research Center of Medical Devices for Cardiovascular Diseases, Ministry of Education, National Clinical Research Center for Cardiovascular Diseases, Beijing, People’s Republic of China; Liverpool Centre for Cardiovascular Science at University of Liverpool, Liverpool John Moores University and Liverpool Heart & Chest Hospital, William Henry Duncan Building, 6 West Derby Street, Liverpool L7 8TX, UK; Tianjin Key Laboratory of Ionic-Molecular Function of Cardiovascular Disease, Department of Cardiology, Tianjin Institute of Cardiology, the Second Hospital of Tianjin Medical University, Tianjin, People’s Republic of China; Department of Cardiovascular Medicine, The Second Affiliated Hospital, Jiangxi Medical College, Nanchang University, Nanchang, Jiangxi, People’s Republic of China; Liverpool Centre for Cardiovascular Science at University of Liverpool, Liverpool John Moores University and Liverpool Heart & Chest Hospital, William Henry Duncan Building, 6 West Derby Street, Liverpool L7 8TX, UK; Department of Cardiology, the First Affiliated Hospital of Chongqing Medical University, Chongqing, People’s Republic of China; Liverpool Centre for Cardiovascular Science at University of Liverpool, Liverpool John Moores University and Liverpool Heart & Chest Hospital, William Henry Duncan Building, 6 West Derby Street, Liverpool L7 8TX, UK; Department of Cardiology, Intermountain Medical Center, Murray, UT, USA; Liverpool Centre for Cardiovascular Science at University of Liverpool, Liverpool John Moores University and Liverpool Heart & Chest Hospital, William Henry Duncan Building, 6 West Derby Street, Liverpool L7 8TX, UK; Department of Cardiovascular and Metabolic Medicine, Institute of Life Course and Medical Sciences, University of Liverpool, William Henry Duncan Building, 6 West Derby Street, Liverpool L7 8TX, UK; Liverpool Centre for Cardiovascular Science at University of Liverpool, Liverpool John Moores University and Liverpool Heart & Chest Hospital, William Henry Duncan Building, 6 West Derby Street, Liverpool L7 8TX, UK; Department of Clinical Medicine, Aalborg University, Selma Lagerløfs Vej 249, Gistrup 9260, Denmark; Medical University of Bialystok, 1 Kilińskiego St, Bialystok 15-089, Poland

**Keywords:** Atrial fibrillation, CABANA trial, Catheter ablation, Early recurrence of atrial tachyarrhythmia, Blanking period

## Abstract

**Aims:**

Early recurrence of atrial tachyarrhythmia (ERAT) after catheter ablation for atrial fibrillation (AF) is common, yet its prognostic significance and implications for the duration of the blanking period remain uncertain. We aimed to evaluate the association between ERAT and post-ablation clinical outcomes, late arrhythmia recurrence, and AF burden, and to explore the optimal duration of the blanking period.

**Methods and results:**

This *post hoc* analysis included 811 patients from the CABANA trial who underwent catheter ablation and had trial-specific electrocardiographic monitoring data available. ERAT was defined as any episode of AF, atrial flutter, or atrial tachycardia lasting >30 s within the 90-day blanking period. The primary outcome was a composite of all-cause mortality, disabling stroke, serious bleeding, or cardiac arrest. Secondary outcomes included cardiovascular hospitalization and late atrial tachyarrhythmia recurrence. During a median 4 years (IQR 2.5–5.2) follow-up, ERAT occurred in 567 patients (69.9%). Patients with ERAT had higher risks of cardiovascular hospitalization [adjusted hazard ratio (aHR) 1.35, 95% confidence interval (CI) 1.07–1.71] and of the composite outcome of all-cause mortality or cardiovascular hospitalization (aHR 1.33, 95% CI 1.07–1.66), but no increased risk of the primary composite outcome. ERAT was independently associated with late atrial tachyarrhythmia recurrence (aHR 1.35, 95% CI 1.07–1.71). Prognostic relevance varied by timing, with the strongest association observed for ERAT occurring between 60 and 90 days after ablation (aHR 1.80, 95% CI 1.16–2.79). Receiver operating characteristic analysis identified 58 days as the optimal threshold for predicting late arrhythmia recurrence (AUC 0.727).

**Conclusion:**

ERAT was associated with higher risks of cardiovascular hospitalization and late atrial tachyarrhythmia recurrence in a time-dependent manner, supporting a shorter blanking period to better identify patients at risk of adverse rhythm outcomes.

## Graphical Abstract

**Graphical Abstract:**
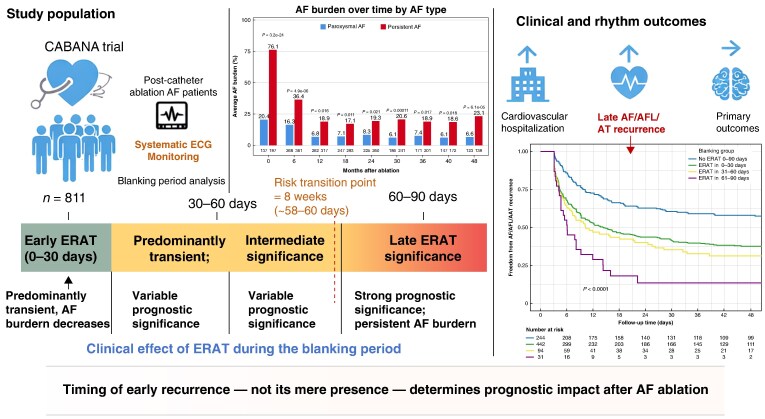

What is new?

- In a large *post hoc* analysis of the CABANA trial, early recurrence of atrial tachyarrhythmia (ERAT) after catheter ablation was associated with an increased risk of cardiovascular hospitalization and late atrial tachyarrhythmia recurrence.
- The prognostic significance of ERAT was strongly time-dependent, with arrhythmias occurring between 60 and 90 days after ablation having the closest association with persistently elevated atrial fibrillation burden and late recurrence.
- Receiver operating characteristic analysis identified an approximately 8-week time threshold after ablation as demonstrating the strongest ability to predict the risk of late recurrence, supporting the suggestion of a shorter blanking period.

## Introduction

Catheter ablation is an established rhythm control strategy for patients with atrial fibrillation (AF), particularly in those with significant symptoms or inadequate response to antiarrhythmic drug therapy.^1–3^ Despite advances in ablation techniques, early recurrence of atrial tachyarrhythmia (ERAT) following catheter ablation remains common and is frequently observed during the early blanking period.^4,5^ These early arrhythmias are traditionally considered transient phenomena, potentially related to ablation-induced myocardial inflammation, oedema, or localized tissue injury, which may resolve as post-procedural healing progresses.^6,7^ Consequently, the clinical significance of ERAT and its relationship with late AF recurrence remains incompletely understood.

To account for these transient post-ablation arrhythmias, the Expert Consensus Statements on Catheter and Surgical Ablation of Atrial Fibrillation recommends blanking period, during which atrial tachyarrhythmias are not considered indicative of ablation failure.^3^ Several studies have demonstrated that ERAT within the blanking period is associated with an increased risk of late AF recurrence.^8–10^ A meta-analysis including six studies reported an overall predictive value of approximately 64.9% for ERAT in identifying late AF recurrence, with substantially higher predictive performance in patients with persistent AF compared with those with paroxysmal AF (81.2% vs. 59.7%).^11^

Beyond the presence of early recurrence, the burden of AF during the blanking period has emerged as an important determinant of long-term rhythm outcomes. In a cohort of 613 patients undergoing continuous rhythm monitoring after catheter ablation, an AF burden exceeding 4.5% within the first 2 months post-ablation was independently associated with late AF recurrence.^12^ Similarly, an AF burden greater than 18% during the previously recommended 90-day blanking period has been identified as a robust predictor of late atrial tachyarrhythmia recurrence.^13^

Against this background, substantial uncertainty persists regarding the most clinically relevant time threshold for defining post-ablation recurrence.^14,15^ Prior studies have suggested that 2 months after catheter ablation may represent an optimal time point for defining AF recurrence,^16^ whereas in patients undergoing cryoballoon pulmonary vein isolation, AF recurrence beyond 30 days post-procedure has been shown to markedly increase the risk of late recurrence.^17^ Accordingly, the optimal duration of the blanking period has become an area of active debate. Reflecting these evolving data, the 2024 EHRA/HRS/APHRS/LAHRS expert consensus statement recommended shortening the blanking period in AF ablation trials from 12 weeks to 8 weeks, while maintaining the principle that recurrences during this interval should not be classified as treatment failure.^3^ However, even with this expert consensus, the optimal timing for defining clinically meaningful post-ablation recurrence remains uncertain, given that existing evidence has been largely based on relatively small studies without implantable or continuous rhythm monitoring.^18^

The CABANA trial (Catheter Ablation vs. Antiarrhythmic Drug Therapy for Atrial Fibrillation) is a large, multicentre, randomized controlled study comparing catheter ablation with drug therapy in patients with AF.^19^ The trial incorporated systematic ECG monitoring, including the *trans*-telephonic monitor (TTM) symptom-driven 2-min recordings, 24-h TTM recordings and continuous Holter recordings for up to 96 h, which could provide detailed characteristics of atrial tachyarrhythmia events. Leveraging data from the CABANA trial, the present study aimed to investigate the associations between AF recurrence during the blanking period and clinical outcomes beyond 90 days, including late AF recurrence and adverse clinical events. In addition, we explored the most appropriate duration of the blanking period by assessing the prognostic value of different post-ablation time thresholds for long-term clinical outcomes.

## Methods

### Data source

The CABANA trial was a multinational, multicentre, open-label randomized clinical trial, the design, rationale, and primary results of which have been reported previously.^19,20^ Patients were enrolled between November 2009 and April 2016 and followed through December 2017. Participants were randomized assigned in a 1:1 ratio to catheter ablation or drug therapy using a centralized, stratified randomization system.

In the ablation group, pulmonary vein isolation was performed in all patients, with additional ablation strategies applied at the discretion of the treating physician. Patients assigned to drug therapy were managed primarily with rate control, with rhythm-control agents or electrical cardioversion used when clinically indicated. Oral anticoagulation was prescribed according to CHA_2_DS_2_-VASc score (≥2). The study protocol was approved by the institutional review board or ethics committee at each participating centre, and all patients provided written informed consent. The trial was conducted in accordance with the Declaration of Helsinki and the CONSORT guidelines. The dataset is available through the National Heart, Lung, and Blood Institute’s Biologic Specimen and Data Repository Information Coordinating Center.

### Study design and population

The study design and patient selection are illustrated in Supplementary material online, *Figure S1*. Among 2204 patients enrolled in the CABANA trial, 853 patients without available CABANA-specific electrocardiographic (ECG) monitoring data were excluded from this analysis. Additionally, 143 patients initially randomized to the drug therapy group who subsequently crossed over to catheter ablation during the trial were included, with follow-up time redefined from the date of ablation.

The final study population comprised 811 patients who underwent catheter ablation and had complete CABANA-specific ECG monitoring data available for analysis.

### ECG monitoring and AF burden assessment

The CABANA trial employed a proprietary ECG monitoring system (Medicomp, Inc., Melbourne, FL, USA), which included symptom-triggered 2-min transtelephonic monitor (TTM) recordings, automatically triggered 24-h TTM recordings for atrial tachyarrhythmia detection, and scheduled up to 96 h continuous ambulatory ECG (Holter) recordings.

During the first year after randomization, 24-h loop recordings were obtained monthly, followed by quarterly recordings thereafter. The Holter monitoring was performed every 6 months to quantify AF burden. AF burden was defined as the percentage of time spent in AF relative to the total duration of Holter monitoring. The detailed information of ECG monitoring was described in Supplementary material online, Appendix.

All ECG recordings were initially reviewed by trained device technicians according to CABANA protocols, followed by adjudication by two independent expert reviewers. Discrepancies were resolved by a third reviewer.

### Study outcomes

The primary outcome was identical to that of the CABANA trial and consisted of a composite of all-cause mortality, disabling stroke, serious bleeding, or cardiac arrest. Secondary outcomes included the following: (i) cardiovascular hospitalization and (ii) a composite of all-cause mortality or cardiovascular hospitalization.

Disabling stroke, including intracranial haemorrhage, was defined as an irreversible neurological deficit result in a modified Rankin Scale score ≥2. Serious bleeding was defined as bleeding accompanied by haemodynamic compromise, requiring surgical intervention or transfusion of at least 3 units of blood.

The ERAT was defined as any episode of AF, atrial flutter (AFL), or atrial tachycardia (AT) lasting >30 s occurring within the 90-day blanking period following catheter ablation. Late recurrence was defined as atrial tachyarrhythmia occurring >90 days after ablation.

### Statistical analysis

Baseline variables with missing values (see Supplementary material online, *Table S1*) were imputed using multiple imputation with chained equations, generating five imputed datasets (*m* = 5) using the ‘mice’ package in R. Continuous variables were non-normally distributed and are presented as median (interquartile range [IQR]), with comparisons performed using the Wilcoxon rank-sum test. Categorical variables are presented as counts and percentages and compared using Pearson’s χ^2^ test or Fisher’s exact test, as appropriate. The definitions of medical history were shown in Supplementary material online, *Table S2*.

Patients were stratified according to the presence or absence of ERAT during the 90-day blanking period. Event rates were compared using Fisher’s exact test. Time-to-first-event analyses for clinical outcomes and late atrial tachyarrhythmia recurrence were performed using Cox proportional hazards models, adjusting for age, sex, AF type, and CHA_2_DS_2_-VASc score. Hazard ratios (HRs) with 95% confidence intervals (CIs) were reported.

Cumulative incidence of clinical events and atrial tachyarrhythmia recurrence was estimated using Kaplan-Meier methods, with between-group differences assessed by the log-rank test. Subgroup analyses were conducted according to age (<65 vs. ≥ 65 years), sex, AF type (paroxysmal vs. persistent), and CHA_2_DS_2_-VASc score, with interaction testing performed using likelihood ratio tests.

Longitudinal changes in AF burden were analysed using linear mixed-effects models with random intercepts to account for repeated Holter measurements. Receiver operating characteristic (ROC) curve analysis was performed to identify the optimal blanking period duration for predicting late atrial tachyarrhythmia recurrence.

A two-sided *P* value <0.05 was considered statistically significant. All analyses were performed using R software (version 4.4.0; R Foundation for Statistical Computing, Vienna, Austria).

## Results

### Baseline characteristics

Baseline characteristics are summarized in *Table 1*. Among the 811 patients included in the analysis, the median age was 68.0 years (IQR 63.0–72.0), 64.5% were male, and 94.0% were White. ERAT during the 90-day blanking period occurred in 567 patients (69.9%).

**Table 1:** Baseline characteristics of patients stratified by ERAT

Characteristics	Total (*n* = 811)	No EART (*n* = 244)	EART (*n* = 567)	*P* value
Age, years	68.0 (63.0, 72.0)	68.0 (64.0, 73.0)	68.0 (63.0, 72.0)	0.037
Male, *n* (%)	523 (64.5)	151 (61.9)	372 (65.6)	0.349
Body mass index, Kg/m^2^	30.3 (26.8, 34.8)	29.1 (26.0, 34.0)	31.0 (27.1, 35.1)	0.008
Systolic blood pressure, mmHg	129.0 (118.0, 140.0)	130.0 (119.0, 141.0)	128.0 (118.0, 139.0)	0.211
Diastolic blood pressure, mmHg	77.0 (70.0, 84.0)	76.0 (69.7, 82.0)	78.0 (70.0, 84.0)	0.118
Pulse rate, bpm	73.0 (62.0, 86.0)	68.0 (60.0, 79.5)	76.0 (64.0, 88.7)	<0.001
Race, *n* (%)				0.193
White	762 (94.0)	228 (93.4)	534 (94.2)	
Black or African American	25 (3.0)	11 (4.5)	14 (2.5)	
Others	24 (3.0)	5 (2.0)	19 (3.4)	
NYHA Class, *n* (%)				0.647
No CHF or Class I	590 (72.8)	173 (70.9)	417 (73.5)	
Class II or greater	221 (27.2)	71 (29.1)	150 (26.5)	
AF severity, *n* (%)				0.072
Class 0	53 (6.5)	22 (9.0)	31 (5.5)	
Class 1	98 (12.1)	33 (13.5)	64 (11.5)	
Class 2	225 (27.7)	71 (29.1)	154 (27.2)	
Class 3	362 (44.6)	106 (43.4)	256 (45.1)	
Class 4	73 (9.0)	12 (4.9)	61 (10.7)	
AF type, *n* (%)				<0.001
Paroxysmal AF	350 (43.2)	132 (54.1)	218 (38.4)	
Persistent AF	396 (48.8)	95 (38.9)	301 (53.1)	
Long-standing persistent AF	65 (8.0)	17 (7.0)	48 (8.5)	
CHA_2_DS_2_-VASc Score	3.0 (2.0, 4.0)	3.0 (2.0, 4.0)	3.0 (2.0, 4.0)	0.684
0–1	125 (16.6)	38 (15.6)	97 (17.1)	
2	207 (25.5)	61 (25.0)	146 (25.7)	
3	232 (28.6)	67 (27.5)	165 (29.1)	
4	136 (16.8)	43 (17.6)	93 (16.4)	
≥ 5	101 (12.6)	35 (14.3)	66 (11.7)	
Anticoagulation status, *n* (%)				0.010
Warfarin	341 (42.0)	90 (36.9)	251 (44.3)	
DOACs^a^	126 (15.5)	38 (15.6)	88 (15.5)	
Medical history				
Coronary artery disease	171 (21.1)	60 (24.6)	111 (19.6)	0.131
Hypertension	620 (76.4)	194 (79.5)	426 (75.1)	0.209
Congestive heart failure	131 (16.2)	33 (13.5)	98 (17.3)	0.219
Diabetes mellitus	219 (27.0)	55 (22.5)	164 (28.9)	0.073
Valve disease	111 (13.7)	30 (12.3)	81 (14.3)	0.519
Oesophageal disease	191 (23.6)	56 (23.0)	135 (23.8)	0.862
Sleep apnoea	240 (29.6)	68 (27.9)	172 (30.3)	0.534
Chronic lung disease	74 (9.1)	20 (8.2)	54 (9.5)	0.639
Cancer	55 (6.8)	12 (4.9)	43 (7.6)	0.218
Renal disease	3 (0.4)	0 (0.0)	3 (0.5)	0.612
Thromboembolic events (peripheral)	33 (4.1)	9 (3.7)	24 (4.2)	0.868
Family history of AF	124 (15.3)	39 (16.0)	85 (15.0)	0.800
History of CVA/TIA	85 (10.5)	25 (10.2)	60 (10.6)	0.985

^a^DOACs included dabigatran, rivaroxaban, apixaban, and edoxaban.

All continuous variables are presented as median (interquartile range) due to non-normal distributions; categorical variables are presented as number (percentage).

AF, atrial fibrillation; CVA, cerebral vascular accident; DOAC, direct oral anticoagulant; ERAT, early recurrence of atrial tachyarrhythmia; NYHA, New York Heart Association; TIA, transient ischaemic attack.

Patients with ERAT were more likely to have persistent or long-standing persistent AF (61.6% vs. 45.9%), had a higher body mass index, and exhibited a slightly higher resting pulse rate compared with patients without ERAT. Mean CHA_2_DS_2_-VASc scores were similar between groups. Median follow-up duration was 4.0 years (IQR 2.5–5.2). Baseline characteristics stratified by time of ERAT are shown in Supplementary material online, *Table S3*.

### AF burden

**Figure 1:**
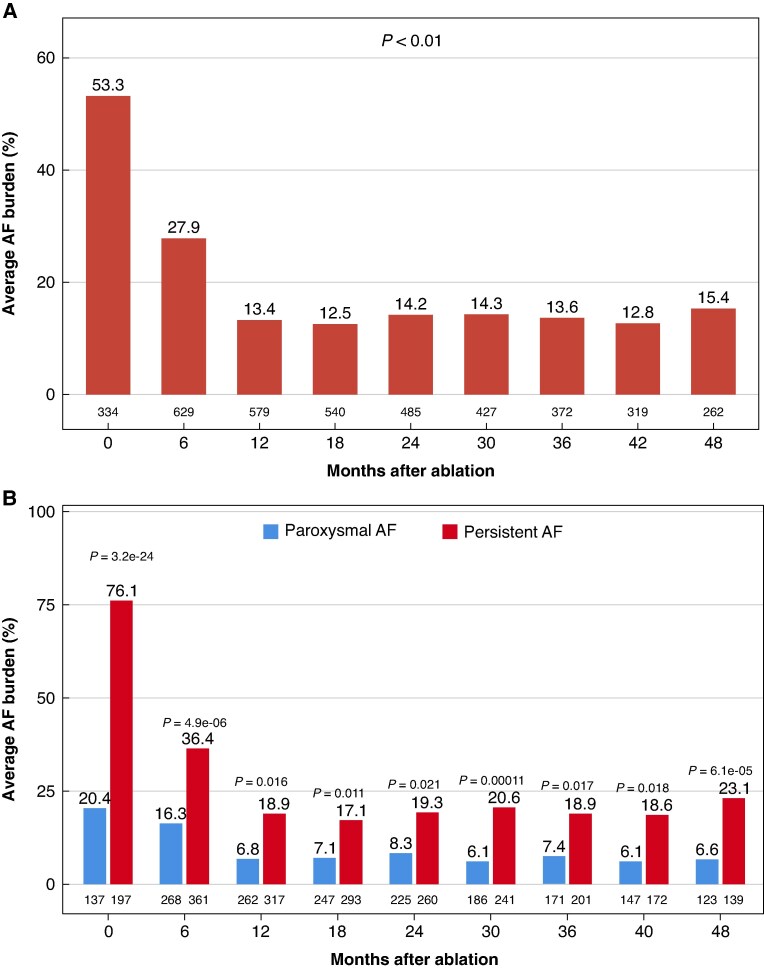
**AF burden assessed at 6-month interval in 811 patients using CABANA ECG 96-h Holter monitors.**  *(A*) AF burden for 811 participants during the 48 months follow-up. *(B*) AF burden according to baseline AF type. AF, atrial fibrillation.

At baseline, AF accounted for a median of 53.3% of total Holter recording time. Following catheter ablation, AF burden declined progressively over time, reaching a low level at approximately 12 months, after which it remained relatively stable (*Figure 1A*). Over 4 years of follow-up, catheter ablation was associated with a sustained and statistically significant reduction in AF burden (*P* < 0.01).

Patients with persistent AF had a significantly higher AF burden at baseline compared with those with non-persistent AF (76.1% vs. 20.4%, *P* < 0.001), and this difference persisted throughout follow-up (*Figure 1B*). At 4 years, median AF burden remained higher in patients with persistent AF than in those with non-persistent AF (23.1% vs. 6.6%, *P* < 0.001).

When stratified by timing of ERAT, patients with ERAT occurring within 0–30 days exhibited the highest baseline AF burden. AF burden declined substantially over time in patients with ERAT occurring within 0–30 and 30–60 days, reaching lowest levels by 12 months (0–30 days: 74.9% to 15.9%, *P* < 0.001; 30–60 days: 37.0% to 15.3%, *P* < 0.01). In contrast, patients with ERAT occurring between 60–90 days demonstrated little reduction in AF burden over follow-up (see Supplementary material online, *Figure S2*), suggesting a distinct arrhythmogenic phenotype.

### Risk of clinical events

During follow-up, a total of 570 composite clinical events were recorded among AF patients who underwent catheter ablation. Patients who experienced ERAT during the 90-day blanking period had a significantly higher incidence of cardiovascular hospitalization compared with those without ERAT (65.6% vs. 44.7%, *P* < 0.001). The incidence of the composite outcome of all-cause mortality or cardiovascular hospitalization was significantly higher in patients with ERAT (66.7% vs. 46.7%, *P* < 0.001).

In contrast, the incidence of the primary composite outcome (all-cause mortality, disabling stroke, serious bleeding, or cardiac arrest), as well as each individual component of the primary outcome, did not differ significantly between patients with and without ERAT (see Supplementary material online, *Table S4*). When patients with ERAT were further stratified according to the timing of ERAT occurrence (0–30 days, 30–60 days, and 60–90 days), similar patterns were observed, with higher rates of cardiovascular hospitalization and all-cause mortality or cardiovascular hospitalization across all ERAT timing subgroups (see Supplementary material online, *Table S5*).

Kaplan–Meier analyses demonstrated a significantly higher cumulative incidence of cardiovascular hospitalization and all-cause mortality or cardiovascular hospitalization in patients with ERAT compared with those without ERAT (both log-rank *P* < 0.001; Supplementary material online, *Figure S3*). No significant difference was observed between groups for the primary composite outcome over time.

In multivariable Cox proportional hazards models adjusted for age, sex, AF type, and CHA_2_DS_2_-VASc score, ERAT during the 90-day blanking period remained independently associated with an increased risk of cardiovascular hospitalization (HR 1.35, 95% CI 1.07–1.71) and of all-cause mortality or cardiovascular hospitalization (HR 1.33, 95% CI 1.07–1.66) (see Supplementary material online, *Table S6*).

When stratified by the timing of ERAT, occurrence of ERAT at 0–30 days (HR 1.84, 95% CI 1.45–2.30), 30–60 days (HR 1.91, 95% CI 1.39–2.61), and 60–90 days (HR 1.77, 95% CI 1.11–2.82) was consistently associated with a significantly higher risk of cardiovascular hospitalization compared with patients without ERAT, as described in *Table 2*.

**Table 2:** HRs comparing the risk of clinical events in patients with and without ERAT across different blanking periods (<30 days, 30–60 days, 60–90 days)

Clinical outcome	aHR (95% CI), *P* value			
	No ERAT	ERAT within 30 days	ERAT at 30–60 days	ERAT at 60–90 days
Primary outcome	*Ref*	1.05 (0.63, 1.75), 0.845	1.19 (0.56, 2.51), 0.647	—
Secondary outcome				
CV hospitalization	*Ref*	1.84 (1.48, 2.30), <0.001	1.91 (1.39, 2.61), <0.001	1.77 (1.11, 2.82), 0.017
All-cause mortality/CV hospitalization	*Ref*	1.81 (1.45, 2.24), <0.001	1.83 (1.34, 2.50), <0.001	1.70 (1.06, 2.70) 0.026
Later AF recurrence	*Ref*	1.32 (1.04, 1.67), 0.023	1.40 (1.02, 1.94), 0.040	1.80 (1.16, 2.79), 0.009
Later AF/AFL/AT recurrence	*Ref*	1.29 (1.03, 1.63), 0.025	1.39 (1.02, 1.92), 0.039	1.73 (1.12, 2.67), 0.013

aHRs and corresponding 95% CIs were estimated using Cox proportional hazards models adjusting for age, sex, AF type, and CHA_2_DS_2_-VASc score. The ‘No ERAT’ group was defined as having no AF/AFL/AT recurrence within 90 days after catheter ablation. This group was used as the reference group for comparisons.

The primary outcome was a composite endpoint including all-cause mortality, disabling stroke, cardiac arrest or serious bleeding events.

Later AF recurrence or Later AF/AFL/AT recurrence was defined as the events occurring after 90-day blanking period of catheter ablation.

AF, atrial fibrillation; AFL, atrial flutter; AT, atrial tachycardia; CI, confidence interval; CV, cardiovascular; ERAT, early recurrence of atrial tachyarrhythmia; HR, hazard ratio.

### Recurrence of atrial tachyarrhythmias after the 90-day blanking period

Over a median follow-up of 4 years, patients who experienced ERAT during the 90-day blanking period had a markedly higher incidence of late AF recurrence compared with those without ERAT (62.8% vs. 32.3%, *P* < 0.001). A similar pattern was observed for the composite endpoint of AF, atrial flutter (AFL), or atrial tachycardia (AT) recurrence (64.4% vs. 34.8%, *P* < 0.001) (see Supplementary material online, *Table S4*).

After adjustment for age, sex, AF type, and CHA_2_DS_2_-VASc score, ERAT during the blanking period was independently associated with an increased risk of late AF recurrence (HR 1.35, 95% CI 1.07–1.71) and composite atrial tachyarrhythmia recurrence (HR 1.33, 95% CI 1.07–1.66) (see Supplementary material online, *Table S5*).

**Figure 2:**
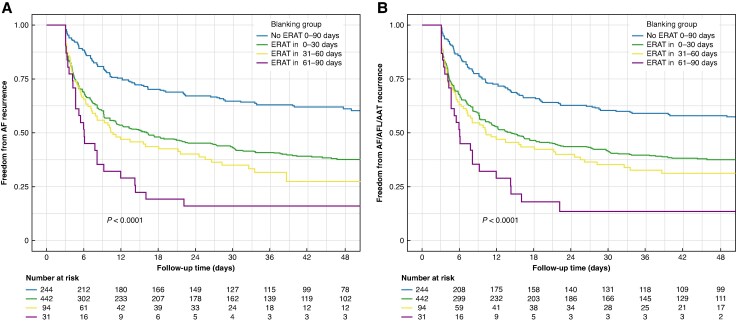
Kaplan–Meier survival curve of freedom from atrial tachyarrhythmia occurring between days 91 and 48 months after catheter ablation on the last occurrence of ERAT during a 90-day blanking period. (*A,B*) AF recurrence and AF/AFL/AT recurrence, separately. ERAT is the early recurrence of atrial tachyarrhythmia during the 90-day blanking period. AF, atrial fibrillation; AFL, atrial flutter; AT, atrial tachycardia; ERAT, early recurrence of atrial tachyarrhythmia.

Kaplan–Meier analyses demonstrated significantly higher cumulative incidence of both AF recurrence and composite atrial tachyarrhythmia recurrence in patients with ERAT, especially for the group of ERAT in 60–90 days (log-rank *P* < 0.001 for both; *Figure 2*).

Notably, patients who experienced ERAT between 60 and 90 days after catheter ablation exhibited the highest risk of late recurrence. Compared with patients without ERAT, ERAT occurring at 60–90 days was associated with an adjusted HR of 1.80 (95% CI 1.16–2.79) for AF recurrence and 1.73 (95% CI 1.12–2.67) for composite atrial tachyarrhythmia recurrence (*Table 2*).

**Figure 3:**
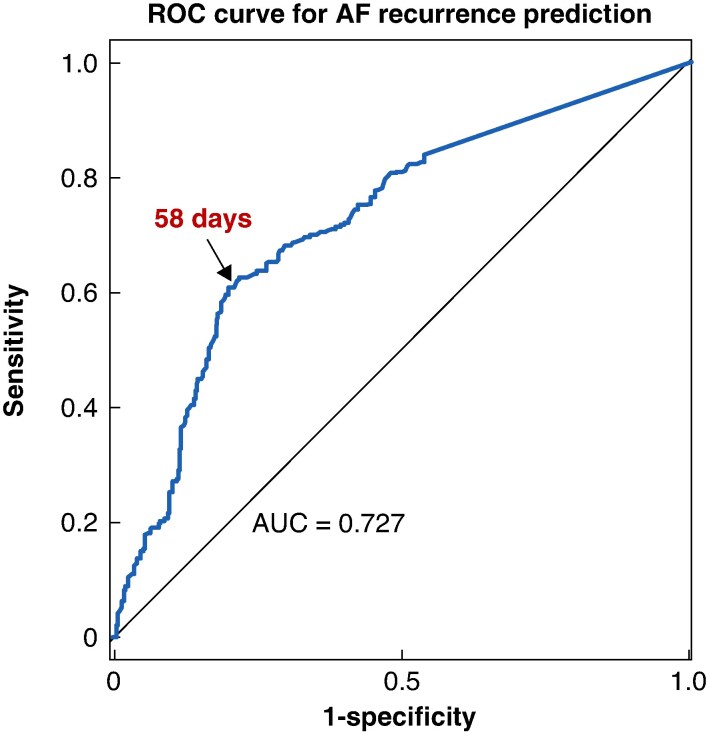
ROC curve for optimal duration of blanking period (AF). AF, atrial fibrillation; AUC, area under the curve; ROC, receiver operating characteristic. Sensitivity: 0.618; Specificity: 0.802.

To further explore the prognostic value of ERAT timing for late AF recurrence, receiver operating characteristic (ROC) analysis was performed. An optimal cut-off of 58 days after catheter ablation was identified for predicting AF recurrence beyond the conventional 90-day blanking period (area under the curve 0.727), with a sensitivity of 0.619 and specificity of 0.802 (*Figure 3*). The ROC result of AF/AFL/AT recurrence was 0.730 and is shown in Supplementary material online, *Figure S4*.

### Sensitivity analysis

To further explore the optimal duration of the blanking period, a sensitivity analysis was performed using a 60-day blanking period. As shown in Supplementary material online, *Tables S7* and *S8*, patients who experienced ERAT within the 60-day blanking period had a similar risk of late AF recurrence (HR 1.20, 95% CI 0.97–1.48) and composite AF/AFL/AT recurrence (HR 0.99, 95% CI 0.81–1.21) compared with those without ERAT.

With respect to clinical outcomes, ERAT occurring within the 60-day blanking period was associated with a higher risk of hospitalization (HR 1.72, 95% CI 1.41–2.11) and of the composite outcome of all-cause mortality or hospitalization (HR 1.69, 95% CI 1.38–2.06), whereas the risk of the primary composite outcome did not differ between groups (HR 1.25, 95% CI 0.76–2.04). Cox regression analyses stratified by the timing of ERAT yielded results consistent with the primary analyses and are presented in Supplementary material online, *Table S9*.

### Subgroup analysis

Subgroup analyses demonstrated no significant interactions between ERAT and age, sex, AF type, or CHA_2_DS_2_-VASc score for either clinical outcomes or late atrial tachyarrhythmia recurrence (all *P*-_for interaction_>0.05) (see Supplementary material online, *Tables S10* and Supplementary material online, *Tables S11*).

## Discussion

In this *post hoc* analysis of the CABANA trial dataset, we investigated the prognostic significance of ERAT following catheter ablation in patients with AF. First, we found that ERAT occurring during the conventional 90-day blanking period was independently associated with an increased risk of cardiovascular hospitalization and late atrial tachyarrhythmia recurrence. Second, the prognostic implications of ERAT varied according to its timing, with ERAT occurring between 60 and 90 days after ablation demonstrating the strongest association with persistently elevated AF burden and late recurrence. Third, ROC analysis also identified 58 days as the optimal cut-off point for predicting late AF recurrence, which is consistent with the recently proposed shorter 8-week blanking period.^3^

Although early atrial tachyarrhythmias have traditionally been considered transient events related to post-ablation inflammation or myocardial injury,^21^ our findings suggest that ERAT is not clinically benign. Patients who experienced ERAT had a significantly higher risk of cardiovascular hospitalization and of the composite outcome of all-cause mortality or cardiovascular hospitalization, even after adjustment for established clinical risk factors. In contrast, ERAT was not associated with an increased risk of the primary composite endpoint, indicating that early recurrence may be more closely related to overall disease burden and healthcare utilization than to irreversible adverse clinical events. Consistent with this interpretation, prior analyses from the CABANA trial demonstrated that catheter ablation leads to substantial improvements in quality of life among symptomatic patients with AF and is more cost-effective than medical therapy,^1,22^ findings that have been corroborated by a meta-analysis of six randomized controlled trial.^23^

Moreover, patients with a higher burden of comorbidities appear to derive even greater quality-of-life benefits from ablation.^24^ Importantly, recurrent atrial tachyarrhythmia is associated with higher AF burden and increased healthcare resource utilization, and patients without recurrence experience greater and more sustained improvements in quality of life than those with recurrent arrhythmia.^25^

The optimal duration of the blanking period after AF ablation remains controversial. Prior work has shown that detection of early atrial tachyarrhythmia within 14 days after ablation has been linked to a more than six-fold increase in cardiovascular hospitalization and cardioversion events during the subsequent 8 weeks.^26^ Notably, extending the blanking period from 30 to 90 days does not appear to reduce the incidence of late AF recurrence.^27^ Consistent with this observation, analyses examining the timing of ERAT have suggested that approximately 64 days may represent an optimal threshold for predicting late recurrence.^16^

However, the evidence from published studies remains heterogeneous. A meta-analysis included seventeen studies indicated that a 4-week blanking period represents the optimal cut-off for defining recurrence.^18^ Differences in ECG monitoring strategies-including monitoring duration, intensity, and modality-may influence the detection of atrial arrhythmia and, consequently, the definition and clinical interpretation of blanking period.^14,28,29^ This ongoing topic has recently been highlighted in a Controversy article, which contrasts the rationale for maintaining a standardized blanking period with arguments supporting a shorter or more individualized approach.^30^ In our study, ERAT occurring beyond approximately 60 days after ablation was associated with persistently elevated AF burden, a higher risk of late recurrence, and increased cardiovascular hospitalization, whereas earlier events appeared to carry less prognostic weight.

Importantly, our results do not support complete elimination of the blanking period. Rather, they suggest that a uniform 90-day window may be overly simplistic and that the clinical significance of ERAT is time-dependent. In line with our ROC analysis identifying a threshold of 58 days, these findings support a more refined, time-adapted interpretation, whereby arrhythmias occurring beyond the second month after ablation may warrant closer clinical attention.

It should be noted that the participants in the CABANA trial were predominantly treated with radiofrequency catheter ablation, and the findings regarding the blanking period may not be directly applicable to pulsed field ablation (PFA), which is based on myocardial-selective irreversible electroporation and has emerged as an alternative method.^31^ Several studies have demonstrated that PFA was noninferior with respect to freedom from adverse clinical outcomes, documented atrial tachyarrhythmia after a 3-month blanking period.^32–34^ PFA appears to induce substantially less inflammatory response and tissue injury, potentially resulting in different patterns of early recurrence.^35–37^ One multicentre observational study reported that ERAT occurring within the conventional 90-day blanking period after PFA was associated with three- to six-fold increased risk of late atrial tachyarrhythmia recurrence.^38^ However, further analyses indicated that ERAT occurring during the second and third months after PFA were associated with a significantly increased risk of late recurrence.^39^

Beyond the presence of ERAT itself, our analysis of serial Holter-derived AF burden provides additional mechanistic insights into the heterogeneous prognostic significance of early recurrence. While patients who experienced ERAT within the first 60 days after ablation showed substantial and sustained reductions in AF burden during follow-up, those with ERAT occurring between 60 and 90 days demonstrated persistently elevated AF burden despite ablation. Prior studies support the relevance of both the timing of recurrence and the magnitude of AF burden. Patients whose first recurrence occurred around the third month after ablation have been shown to experience a significantly higher AF burden compared with those recurring between 4 and 12 months or after 12 months.^40^

AF burden has also emerged as a strong predictor of late recurrence. Prolonged electrocardiographic monitoring during the second and third months after ablation has demonstrated that an atrial tachyarrhythmia burden of ≥23 min per day is associated with an approximately seven-fold increase in recurrence risk.^41^ Similarly, continuous rhythm monitoring using smartphone-based devices has shown that an AF burden exceeding 18% during the blanking period is associated with a significantly higher risk of AF recurrence within 18 months.^13^ Conversely, lower AF burden within the first 6 months after ablation is associated with reduced risks of all-cause mortality and heart failure.^42^ Taken together, these observations suggest that AF burden may more accurately capture the clinical impact of catheter ablation on arrhythmia control and symptoms, and may serve as a valuable prognostic marker beyond the binary classification of recurrence.^43^

In the present study, we further observed that patients with persistent AF consistently exhibited a higher AF burden after catheter ablation compared with those with paroxysmal AF. This finding is consistent with prior reports demonstrating higher recurrence rates and less durable rhythm control after ablation in patients with persistent AF.^44,45^ Persistent AF is typically associated with a greater burden of comorbidities and more advanced atrial structural and electrical remodelling, including left atrial enlargement, increased atrial fibrosis, and conduction abnormalities.^46,47^ These features can be interpreted within the framework of atrial cardiomyopathy, which encompasses structural, functional, and electrophysiological alterations of the atrium and is increasingly recognized as key substrate underlying AF persistence, ablation failure and late AF recurrence.^48^ Moreover, persistent AF often represents a later stage in the natural history of the disease, in which irreversible atrial substrate alterations may limit the long-term ability of catheter ablation to achieve sustained reductions in AF burden.^49^ In this contest, the higher AF burden observed in patients with persistent AF in our study may reflect a more advanced stage of atrial cardiomyopathy. These observations support the potential benefit of performing catheter ablation earlier in the course of AF. Intervention at the paroxysmal stage may delay or prevent progression to persistent AF, reduce long-term AF burden, and potentially mitigate AF-related adverse clinical outcome.^50,51^

### Strengths and limitations

From a clinical perspective, our findings suggest that early post-ablation atrial arrhythmias should not be uniformly dismissed as benign, particularly when occurring beyond the first 2 months after ablation. Patients with later ERAT may benefit from intensified rhythm monitoring and targeted risk factor modification. Several limitations should be acknowledged. This was a *post hoc* analysis, and the impact of specific ablation techniques was not examined in our analysis, which may influence the AF recurrence and long-term prognosis.^52,53^ AF burden was assessed using intermittent Holter monitoring rather than continuous implantable devices, which may result in underestimation of the true arrhythmia burden, particularly for asymptomatic episodes.^54–57^ Finally, residual confounding cannot be excluded, such as the use of antiarrhythmic drugs following catheter ablation.^58^ Nonetheless, the use of rigorously adjudicated ECG data from a large randomized trial strengthens the robustness of our findings.

## Conclusion

In this *post hoc* analysis of the CABANA trial dataset, ERAT after catheter ablation was associated with a higher risk of cardiovascular hospitalizations and subsequent arrhythmia recurrence. The prognostic relevance of early recurrence differed by timing, with later events showing a much closer relationship to increased AF burden and long-term recurrence.

## Perspectives

### Competency in patient care and procedural skills

Early recurrence of atrial tachyarrhythmia after catheter ablation should not be uniformly interpreted as a benign or transient phenomenon. This study demonstrates that the clinical implications of early recurrence are time-dependent, with arrhythmias occurring later during the conventional 90-day blanking period being more closely associated with sustained atrial fibrillation burden and subsequent recurrence. Awareness of these temporal patterns may help clinicians refine post-ablation surveillance, patient counselling, and risk stratification, rather than relying solely on a fixed 90-day definition of treatment success or failure.

### Translational outlook

Although early post-ablation arrhythmias have traditionally been attributed to transient inflammatory or procedural effects, the present findings suggest that their prognostic significance varies according to timing. Further prospective studies using continuous rhythm monitoring are needed to validate optimal time thresholds for defining the blanking period and to determine whether time-adapted management strategies can improve long-term outcomes. In addition, as newer ablation technologies such as PFA are increasingly adopted, future research should examine whether patterns of early recurrence and their clinical implications differ across ablation modalities.

## Supplementary Material

euag173_Supplementary_Data

## Data Availability

The data used in this analysis were obtained from the CABANA trial through the National Heart, Lung, and Blood Institute’s Biologic Specimen and Data Repository Information Coordinating Center (BioLINCC) under a data use agreement. The authors were not involved in the design or conduct of the original trial. Access to the CABANA dataset may be requested through the BioLINCC website (https://biolincc.nhlbi.nih.gov).
