# Supplementary material for: Early recurrence of atrial tachyarrhythmia after catheter ablation and its associations with clinical outcomes, atrial fibrillation burden, and blanking period duration: a post hoc analysis of the CABANA trial
Source: Europace. 2026 Jul 7;28(7):euag173. doi: 10.1093/europace/euag173 (PMC13385998; doi:10.1093/europace/euag173)
Supplement: euag173_Supplementary_Data [file euag173_supplementary_data.docx]

**Early recurrence of atrial tachyarrhythmia after catheter ablation and its associations with clinical outcomes, atrial fibrillation burden, and blanking period duration: a post hoc analysis of the CABANA trial**

**Running title**: Blanking period and AF

**Supplementary Materials**

[Appendix. Detailed description of atrial arrhythmia events monitoring 3](#_Toc218439571)

[Supplementary Table S1. Proportions of missing values for baseline characteristics. 4](#_Toc218439572)

[Supplementary Table S2. Comorbidity definitions according to the electronic Case Report Form of CABANA trial. 5](#_Toc218439573)

[Supplementary Table S3. Baseline characteristics of patients stratified by the timing of ERAT in blanking period. 6](#_Toc218439574)

[Supplementary Table S4. Clinical outcomes of patients with catheter ablation 9](#_Toc218439575)

[Supplementary Table S5. Clinical outcomes of patients with catheter ablation according to the timing of ERAT in blanking period. 10](#_Toc218439576)

[Supplementary Table S6. HRs comparing the risk of clinical events in patients with and without ERAT. 11](#_Toc218439577)

[Supplementary Table S7. Clinical outcomes of patients with catheter ablation according to the timing of ERAT in 60-day blanking period. 12](#_Toc218439578)

[Supplementary Table S8. HRs comparing the risk of clinical events in patients with and without ERAT during the 60-day blanking period. 13](#_Toc218439579)

[Supplementary Table S9. HRs comparing the risk of clinical events in patients with and without ERAT across different blanking periods (<30 days, 30-60 days) 14](#_Toc218439580)

[Supplementary Table S10. Subgroup analysis of the primary and secondary outcomes in the patients with catheter ablation. 15](#_Toc218439581)

[Supplementary Table S11. Subgroup analysis of the tachycardia atrial events in the patients with catheter ablation. 17](#_Toc218439582)

[Supplementary Figure S1. The flowchart of this study. 18](#_Toc218439583)

[Supplementary Figure S2. The atrial fibrillation burden of participant accepted catheter ablation according to timing of ERAT during the 48-months follow-up. 19](#_Toc218439584)

[Supplementary Figure S3. Cumulative clinical events curve in AF patients with different timing of ERAT. 20](#_Toc218439585)

[Supplementary Figure S4. Receiver operating characteristic (ROC) curve for optimal duration of blanking period (AF/AFL/AT). 21](#_Toc218439586)

Appendix. Detailed description of atrial arrhythmia events monitoring

Event monitors were programmed to automatically detect and capture atrial arrhythmias as they occurred. The monitoring algorithm was designed to record the longest atrial arrhythmia episode within any single 24-hour period, with a maximum recording duration of up to two minutes (120 seconds). For each detected event, the monitor provided the maximum and minimum heart rates across all recorded episodes. The duration of individual recordings varied according to the detected arrhythmia length and included segments of 15, 30, 45, 60, 90, or 120 seconds. For atrial arrhythmia episodes lasting longer than two minutes, the event monitor continued recording additional segments, including new maximum and minimum heart rate events if they occurred.

Supplementary Table S1. Proportions of missing values for baseline characteristics.

| **Variable** | **Missing, n (%)** |
| --- | --- |
| Body mass index | 11 (1.35) |
| Systolic blood pressure | 6 (0.74) |
| Diastolic blood pressure | 9 (1.11) |
| Pulse rate | 2 (0.25) |
| Atrial fibrillation severity | 2 (0.25) |
| New York Heart Association Functional Classification | 5 (0.62) |

Supplementary Table S2. Comorbidity definitions according to the electronic Case Report Form of CABANA trial.

| **Comorbidity** | **Definition** |
| --- | --- |
| Coronary artery disease | Defined as including MI, CABG, PCI, or previously diagnosed CAD |
| Hypertension | Defined as systolic blood pressure over 140 mmHg or diastolic blood pressure over 90 mmHg |
| Congestive heart failure | No formal definition provided |
| Diabetes mellitus | Defined as fasting plasma glucose over 126 mg/dL |
| Valve disease | Defined as the presence of valvular stenosis or insufficiency (regurgitation) |
| Esophageal disease | No formal definition provided |
| Sleep apnea | No formal definition provided |
| Chronic lung disease | No formal definition provided |
| Cancer | No formal definition provided |
| Renal disease | Defined as the presence of renal disease and/or current or anticipated dialysis |
| Peripheral thromboembolic events | No formal definition provided |
| History of CVA/TIA | No formal definition provided |

Abbreviations: CABG, coronary artery bypass grafting; CAD, coronary artery disease; CVA, cerebrovascular accident; MI, myocardial infarction; PCI, percutaneous coronary intervention; TIA, transient ischemic attack.

Supplementary Table S3. Baseline characteristics of patients stratified by the timing of ERAT in blanking period.

| **Characteristics** | **Total**  **(N = 811)** | **No EART**  **(N = 244)** | **ERAT within 30 days (N = 442)** | **ERAT at 30-60 days (N = 94)** | **ERAT at 60-90 days (N = 31)** | **P value** |
| --- | --- | --- | --- | --- | --- | --- |
| Age, years | 68.0 (63.0, 72.0) | 68.0 (64.0, 73.0) | 68.0 (62.0, 72.0) | 67.0 (64.2, 70.0) | 68.0 (60.5, 70.5) | 0.142 |
| Male, n (%) | 523 (64.5) | 151 (61.9) | 288 (65.2) | 62 (66.0) | 9 (29.0) | 0.694 |
| Body mass index, Kg/m^2^ | 30.3 (26.8, 34.8) | 29.1 (26.0, 34.0) | 31.1 (27.2, 35.0) | 30.1 (26.9, 34.6) | 31.8 (28.7, 38.9) | 0.023 |
| Systolic blood pressure, mmHg | 129.0 (118.0, 140.0) | 130.0 (119.0, 141.0) | 128.0 (118.0, 140.0) | 129.0 (118.2, 138.0) | 130.0 (120.0, 137.0) | 0.584 |
| Diastolic blood pressure, mmHg | 77.0 (70.0, 84.0) | 76.0 (69.7, 82.0) | 78.0 (70.0, 84.0) | 77.0 (69.0, 85.0) | 74.0 (68.5, 84.0) | 0.306 |
| Pulse rate, bpm | 73.0 (62.0, 86.0) | 68.0 (60.0, 79.5) | 76.0 (65.0, 90.0) | 74.5 (62.2, 87.8) | 71.0 (60.5, 84.0) | <0.001 |
| Race, n (%) |  |  |  |  |  | 0.470 |
| White | 762 (94.0) | 228 (93.4) | 415 (93.9) | 88 (93.6) | 31 (100.0) |  |
| Black or African American | 25 (3.0) | 11 (4.5) | 12 (2.7) | 2 (2.1) | 0 (0.0) |  |
| Others | 24 (3.0) | 5 (2.0) | 15 (3.4) | 4 (4.3) | 0 (0.0) |  |
| NYHA Class, n (%) |  |  |  |  |  | 0.930 |
| No CHF or Class I | 590 (72.8) | 173 (70.9) | 318 (71.9) | 74 (78.7) | 25 (80.6) |  |
| Class II or greater | 221 (27.2) | 71 (29.1) | 124 (28.1) | 20 (21.3) | 6 (19.4) |  |
| AF severity, n (%) |  |  |  |  |  | 0.197 |
| Class 0 | 53 (6.5) | 22 (9.0) | 20 | 10 (10.6) | 2 (6.5) |  |
| Class 1 | 98 (12.1) | 33 (13.5) | 49 (11.1) | 11 (11.7) | 5 (16.1) |  |
| Class 2 | 225 (27.7) | 71 (29.1) | 128 (29.0) | 20 (21.3) | 6 (19.4) |  |
| Class 3 | 362 (44.6) | 106 (43.4) | 196 (44.3) | 44 (46.8) | 16 (51.6) |  |
| Class 4 | 73 (9.0) | 12 (4.9) | 49 (11.1) | 9 (9.6) | 0 (0.0) |  |
| AF type, n (%) |  |  |  |  |  | <0.001 |
| Paroxysmal AF | 350 (43.2) | 132 (54.1) | 157 (35.5) | 45 (47.9) | 16 (51.6) |  |
| Persistent AF | 396 (48.8) | 95 (38.9) | 244 (55.2) | 43 (45.7) | 45.2 (45.2) |  |
| Long-standing persistent AF | 65 (8.0) | 17 (7.0) | 41 (9.3) | 6 (6.4) | 1 (3.2) |  |
| CHA_2_DS_2_-VASc Score | 3.0 (2.0, 4.0) | 3.0 (2.0, 4.0) | 3.0 (2.0, 4.0) | 3.0 (2.0, 4.0) | 3.0 (2.0, 4.0) | 0.831 |
| 0-1 | 125 (16.6) | 38 (15.6) | 78 (17.7) | 14 (14.9) | 5 (16.1) |  |
| 2 | 207 (25.5) | 61 (25.0) | 105 (23.8) | 28 (29.8) | 21 (68.7) |  |
| 3 | 232 (28.6) | 67 (27.5) | 130 (29.4) | 27 (28.7) | 3 (9.7) |  |
| 4 | 136 (16.8) | 43 (17.6) | 32 (7.2) | 13 (13.8) | 2 (6.5) |  |
| ≥ 5 | 101 (12.6) | 35 (14.3) | 52 (11.8) | 2 (2.1) | 0 (0.0) |  |
| Anticoagulation status, n (%) |  |  |  |  |  | 0.008 |
| Warfarin | 341 (42.0) | 90 (36.9) | 209 (47.3) | 28 (29.8) | 14 (45.2) |  |
| DOACs^a^ | 126 (15.5) | 38 (15.6) | 67 (15.2) | 15 (16.0) | 6 (19.3) |  |
| Medical history |  |  |  |  |  |  |
| Coronary artery disease | 171 (21.1) | 60 (24.6) | 89 (20.1) | 15 (16.0) | 7 (22.6) | 0.323 |
| Hypertension | 620 (76.4) | 194 (79.5) | 330 (74.7) | 73 (77.7) | 23 (72.4) | 0.473 |
| Congestive heart failure | 131 (16.2) | 33 (13.5) | 78 (17.6) | 17 (18.1) | 3 (9.7) | 0.361 |
| Diabetes mellitus | 219 (27.0) | 55 (22.5) | 123 (27.8) | 30 (31.9) | 33 (35.5) | 0.170 |
| Valve disease | 111 (13.7) | 30 (12.3) | 67 (15.2) | 10 (10.6) | 4 (12.9) | 0.600 |
| Esophageal disease | 191 (23.6) | 56 (23.0) | 108 (24.4) | 18 (19.1) | 9 (29.0) | 0.649 |
| Sleep apnea | 240 (29.6) | 68 (27.9) | 133 (30.1) | 27 (28.7) | 12 (38.7) | 0.651 |
| Chronic lung disease | 74 (9.1) | 20 (8.2) | 45 (10.2) | 5 (5.3) | 4 (12.9) | 0.402 |
| Cancer | 55 (6.8) | 12 (4.9) | 35 (7.9) | 8 (8.5) | 0 (0.0) | 0.175 |
| Renal disease | 3 (0.4) | 0 (0.0) | 2 (0.5) | 1 (1.1) | 0 (0.0) | 0.502 |
| Thromboembolic events (peripheral) | 33 (4.1) | 9 (3.7) | 21 (4.8) | 3 (3.2) | 0 (0.0) | 0.555 |
| Family history of AF | 124 (15.3) | 39 (16.0) | 66 (14.9) | 14 (14.9) | 5 (16.1) | 0.983 |
| History of CVA/TIA | 85 (10.5) | 25 (10.2) | 50 (11.3) | 9 (9.6) | 1 (3.2) | 0.879 |

^a^DOACs included dabigatran, rivaroxaban, apixaban, and edoxaban.

All continuous variables are presented as median (interquartile range) due to non-normal distributions; categorical variables are presented as number (percentage).

Abbreviations: AF, atrial fibrillation; CVA, cerebral vascular accident; DOAC, direct oral anticoagulant; NYHA, New York Heart Association; TIA, transient ischemic attack.

Supplementary Table S4. Clinical outcomes of patients with catheter ablation

| **Clinical Outcome** | **No ERAT**  **N, (%)** | **ERAT**  **N, (%)** | ***P* value** |
| --- | --- | --- | --- |
| **Primary Outcome** | 22 (9.0) | 56 (9.9) | 0.703 |
| **Components of primary outcome** |  |  |  |
| All-cause death | 10 (4.1) | 36 (6.3) | 0.204 |
| Disabling stroke | 1 (0.4) | 2 (0.4) | 0.902 |
| Cardiac arrest | 2 (0.8) | 2 (0.4) | 0.384 |
| Serious bleeding | 12 (4.9) | 24 (4.2) | 0.664 |
| **Secondary outcome** |  |  |  |
| CV hospitalisation | 109 (44.7) | 372 (65.6) | <0.001 |
| All-cause mortality/CV hospitalisation | 114 (46.7) | 378 (66.7) | <0.001 |
| **Later AF recurrence** | 79 (32.3) | 356 (62.8) | <0.001 |
| **Later AF/AFL/AT recurrence** | 85 (34.8) | 363 (64.4) | <0.001 |

The primary outcome was a composite endpoint including all-cause mortality, disabling stroke, cardiac arrest or serious bleeding events.

Later AF recurrence or Later AF/AFL/AT recurrence was defined as the events occurring after 90-day blanking period of catheter ablation.

Abbreviations: AF, atrial fibrillation; AFL, atrial flutter; AT, atrial tachycardia; CV, cardiovascular; ERAT, early recurrence of atrial tachyarrhythmia.

Supplementary Table S5. Clinical outcomes of patients with catheter ablation according to the timing of ERAT in blanking period.

| **Clinical Outcome** | **No ERAT**  **N, (%)** | **ERAT within 30 days**  **N, (%)** | **ERAT at 30-60 days**  **N, (%)** | **ERAT at 60-90 days**  **N, (%)** | ***P* value** |
| --- | --- | --- | --- | --- | --- |
| Total | 244 | 442 | 94 | 31 |  |
| **Primary Outcome** | 22 (9.0) | 46 (10.4) | 10 (10.6) | 0 (0.0) | 0.280 |
| **Components of primary outcome** |  |  |  |  |  |
| All-cause death | 10 (4.1) | 33 (7.5) | 3 (3.2) | 0 (0.0) | 0.081 |
| Disabling stroke | 1 (0.4) | 1 (0.2) | 1 (3.2) | 0 (0.0) | 0.659 |
| Cardiac arrest | 2 (0.8) | 1 (0.2) | 1 (3.2) | 0 (0.0) | 0.583 |
| Serious bleeding | 12 (4.9) | 19 (4.3) | 5 (5.3) | 0 (0.0) | 0.623 |
| **Secondary outcome** |  |  |  |  |  |
| CV hospitalisation | 109 (44.7) | 290 (65.6) | 61 (64.9) | 21 (67.7) | <0.001 |
| All-cause mortality/CV hospitalisation | 114 (46.7) | 296 (67.5) | 61 (64.9) | 21 (67.7) | <0.001 |
| **Later AF recurrence** | 79 (32.3) | 266 (60.2) | 64 (68.1) | 26 (83.9) | <0.001 |
| **Later AF/AFL/AT recurrence** | 85 (34.8) | 273 (61.8) | 64 (68.1) | 26 (83.9) | <0.001 |

The primary outcome was a composite endpoint including all-cause mortality, disabling stroke, cardiac arrest or serious bleeding events.

Later AF recurrence or Later AF/AFL/AT recurrence was defined as the events occurring after 90-day blanking period of catheter ablation.

Abbreviations: AF, atrial fibrillation; AFL, atrial flutter; AT, atrial tachycardia; CV, cardiovascular; ERAT, early recurrence of atrial tachyarrhythmia.

Supplementary Table S6. HRs comparing the risk of clinical events in patients with and without ERAT.

| **Clinical Outcome** | **Model 1** | | **Model 2** | |
| --- | --- | --- | --- | --- |
|  | **HR (95% CI)** | ***P* Value** | **HR (95% CI)** | ***P* Value** |
| **Primary Outcome** | 1.01 (0.62, 1.66) | 0.957 | 1.10 (0.67, 1.81) | 0.695 |
| **Secondary outcome** |  |  |  |  |
| CV hospitalisation | 1.83 (1.48, 2.27) | <0.001 | 1.85 (1.49, 2.29) | <0.001 |
| All-cause mortality/CV hospitalisation | 1.78 (1.45, 2.20) | <0.001 | 1.80 (1.46, 2.23) | <0.001 |
| **Later AF recurrence** | 1.36 (1.08, 1.71) | 0.009 | 1.35 (1.07, 1.71) | 0.010 |
| **Later AF/AFL/AT recurrence** | 1.38 (1.07, 1.67) | 0.009 | 1.33 (1.07, 1.66) | 0.011 |

Model 1: Univariable model.

Model 2: Adjusted by age, sex, AF type and CHA_2_DS_2_-VASc score.

HRs and corresponding 95% CIs were estimated using Cox proportional hazards models.

The primary outcome was a composite endpoint including all-cause mortality, disabling stroke, cardiac arrest or serious bleeding events.

Later AF recurrence or Later AF/AFL/AT recurrence was defined as the events occurring after 90 days of catheter ablation.

Abbreviations: AF, atrial fibrillation; AFL, atrial flutter; AT, atrial tachycardia; CI, confidence interval; CV, cardiovascular; ERAT, early recurrence of atrial tachyarrhythmia; HR, hazard ratio.

Supplementary Table S7. Clinical outcomes of patients with catheter ablation according to the timing of ERAT in 60-day blanking period.

| **Clinical Outcome** | **No ERAT**  **N, (%)** | **ERAT within 30 days**  **N, (%)** | **ERAT at 30-60 days**  **N, (%)** | ***P* value** |
| --- | --- | --- | --- | --- |
| Total | 275 | 442 | 94 |  |
| **Primary Outcome** | 22 (8.0) | 46 (10.4) | 10 (10.6) | 0.703 |
| **Secondary outcome** |  |  |  |  |
| CV hospitalisation | 130 (47.3) | 290 (65.6) | 61 (64.9) | <0.001 |
| All-cause mortality/CV hospitalisation | 135 (49.1) | 296 (67.0) | 61 (64.9) | <0.001 |
| **Later AF recurrence** | 122 (44.4) | 266 (60.2) | 64 (68.1) | <0.001 |
| **Later AF/AFL/AT recurrence** | 133 (48.4) | 273 (61.8) | 62 (66.0) | <0.001 |

The primary outcome was a composite endpoint including all-cause mortality, disabling stroke, cardiac arrest or serious bleeding events.

Later AF recurrence or Later AF/AFL/AT recurrence was defined as the events occurring after 60-day blanking period of catheter ablation.

Abbreviations: AF, atrial fibrillation; AFL, atrial flutter; AT, atrial tachycardia; CV, cardiovascular; ERAT, early recurrence of atrial tachyarrhythmia.

Supplementary Table S8. HRs comparing the risk of clinical events in patients with and without ERAT during the 60-day blanking period.

| **Clinical Outcome** | **Model 1** | | **Model 2** | |
| --- | --- | --- | --- | --- |
|  | **HR (95% CI)** | ***P* Value** | **HR (95% CI)** | ***P* Value** |
| **Primary Outcome** | 1.23 (0.75, 2.01) | 0.412 | 1.25 (0.76, 2.04) | 0.383 |
| **Secondary outcome** |  |  |  |  |
| CV hospitalisation | 1.71 (1.40, 2.10) | <0.001 | 1.72 (1.41, 2.11) | <0.001 |
| All-cause mortality/CV hospitalisation | 1.68 (1.38, 2.03) | <0.001 | 1.69 (1.38, 2.06) | <0.001 |
| **Later AF recurrence** | 1.22 (0.98, 1.51) | 0.067 | 1.20 (0.97, 1.48) | 0.088 |
| **Later AF/AFL/AT recurrence** | 1.00 (0.81, 1.23) | 0.918 | 0.99 (0.81, 1.21) | 0.983 |

Model 1: Univariable model.

Model 2: Adjusted by age, sex, AF type and CHA_2_DS_2_-VASc score.

HRs and corresponding 95% CIs were estimated using Cox proportional hazards models.

The primary outcome was a composite endpoint including all-cause mortality, disabling stroke, cardiac arrest or serious bleeding events.

Later AF recurrence or Later AF/AFL/AT recurrence was defined as the events occurring after 60 days of catheter ablation.

Abbreviations: AF, atrial fibrillation; AFL, atrial flutter; AT, atrial tachycardia; CI, confidence interval; CV, cardiovascular; ERAT, early recurrence of atrial tachyarrhythmia; HR, hazard ratio.

Supplementary Table S9. HRs comparing the risk of clinical events in patients with and without ERAT across different blanking periods (<30 days, 30-60 days)

| **Clinical Outcome** | **aHR (95% CI), *P* value** | | |
| --- | --- | --- | --- |
|  | **No ERAT** | **ERAT within 30 days** | **ERAT at 30-60 days** |
| **Primary Outcome** | Ref | 1.18 (0.71, 1.96), 0.531 | 1.40 (0.66, 2.96),0.377 |
| **Secondary outcome** |  |  |  |
| CV hospitalisation | Ref | 1.70 (1.39, 2.10), <0.001 | 1.76 (1.30, 2.38), <0.001 |
| All-cause mortality/CV hospitalisation | Ref | 1.68 (1.37, 2.06), <0.001 | 1.72 (1.27, 2.32), <0.001 |
| **Later AF recurrence** | Ref | 1.21 (0.97, 1.51), 0.089 | 1.27 (0.93, 1.73), 0.132 |
| **Later AF/AFL/AT recurrence** | Ref | 1.00 (0.81, 1.23), 0.991 | 1.01 (0.74, 1.36), 0.966 |

aHRs and corresponding 95% CIs were estimated using Cox proportional hazards models adjusting for age, sex, AF type and CHA2DS2-VASc score. The “No ERAT” group was defined as having no AF/AFL/AT recurrence within 60 days after catheter ablation. This group was used as the reference group for comparisons.

The primary outcome was a composite endpoint including all-cause mortality, disabling stroke, cardiac arrest or serious bleeding events.

Later AF recurrence or Later AF/AFL/AT recurrence was defined as the events occurring after 60-day blanking period of catheter ablation.

Abbreviations: AF, atrial fibrillation; AFL, atrial flutter; AT, atrial tachycardia; CI, confidence interval; CV, cardiovascular; ERAT, early recurrence of atrial tachyarrhythmia; HR, hazard ratio.

Supplementary Table S10. Subgroup analysis of the primary and secondary outcomes in the patients with catheter ablation.

| **Clinical outcome** | **Subgroup** | **aHR (95% CI)** | ***P* value** | **Interaction *P* value** |
| --- | --- | --- | --- | --- |
| Primary outcome | **Age** |  |  |  |
|  | <65 years | 0.41 (0.09, 1.92) | 0.260 | 0.211 |
|  | ≥65 years | 1.12 (0.66, 1.91) | 0.656 |  |
|  | **Sex** |  |  |  |
|  | Female | 0.67 (0.31, 1.48) | 0.330 | 0.163 |
|  | Male | 1.38 (0.71, 2.71) | 0.344 |  |
|  | **AF type** |  |  |  |
|  | Paroxysmal AF | 1.01 (0.46, 2.24) | 0.980 | 0.865 |
|  | Persistent AF | 1.04 (0.55, 1.98) | 0.908 |  |
|  | **CHA_2_DS_2_-VASc** |  |  |  |
|  | Low | 1.46 (0.30, 7.06) | 0.634 | 0.893 |
|  | High | 1.05 (0.63, 1.78) | 0.842 |  |
| CV hospitalisation | **Age** |  |  |  |
|  | <65 years | 2.09 (1.31, 3.31) | 0.002 | 0.399 |
|  | ≥65 years | 1.72 (1.34, 2.19) | <0.001 |  |
|  | **Sex** |  |  |  |
|  | Female | 1.74 (1.23, 2.47) | 0.002 | 0.712 |
|  | Male | 1.84 (1.40, 2.43) | <0.001 |  |
|  | **AF type** |  |  |  |
|  | Paroxysmal AF | 2.84 (2.02, 3.98) | <0.001 | 0.494 |
|  | Persistent AF | 1.87 (1.09, 3.23) | 0.023 |  |
|  | **CHA_2_DS_2_-VASc** |  |  | 0.277 |
|  | Low | 2.53 (1.36, 4.73) | 0.003 |  |
|  | High | 1.73 (1.41, 2.23) | <0.001 |  |
| All-cause mortality or CV hospitalisation | **Age** |  |  |  |
|  | <65 years | 1.85 (1.20, 2.88) | 0.006 | 0.685 |
|  | ≥65 years | 1.72 (1.35, 2.20) | <0.001 |  |
|  | **Sex** |  |  |  |
|  | Female | 1.72 (1.22, 2.43) | 0.002 | 0.843 |
|  | Male | 1.78 (1.36, 2.34) | <0.001 |  |
|  | **AF type** |  |  |  |
|  | Paroxysmal AF | 2.74 (1.98, 3.83) | <0.001 | 0.459 |
|  | Persistent AF | 1.80 (1.06, 3.08) | 0.029 |  |
|  | **CHA_2_DS_2_-VASc** |  |  |  |
|  | Low | 2.23 (1.24, 3.99) | 0.007 | 0.445 |
|  | High | 1.75 (1.39, 2.19) | <0.001 |  |

High CHA_2_DS₂-VASc risk was defined as a score ≥2. The group which was defined as having no ERAT after 90-day blanking period of catheter ablatio was used as the reference group for all comparisons.

Abbreviations: aHR, adjusted hazard ratio; AF, atrial fibrillation; CI, confidence interval; CV, cardiovascular.

Supplementary Table S11. Subgroup analysis of the tachycardia atrial events in the patients with catheter ablation.

| **Atrial event** | **Subgroup** | **aHR (95% CI)** | ***P* value** | **Interaction *P* value** |
| --- | --- | --- | --- | --- |
| AF | **Age** |  |  |  |
|  | <65 years | 1.63 (1.06, 2.52) | 0.027 | 0.246 |
|  | ≥65 years | 2.19 (1.70, 2.84) | <0.001 |  |
|  | **Sex** |  |  |  |
|  | Female | 2.00 (1.40, 2.87) | <0.001 | 0.972 |
|  | Male | 2.01 (1.51, 2.67) | <0.001 |  |
|  | **AF type** |  |  |  |
|  | Paroxysmal AF | 2.09 (1.53, 2.86) | <0.001 | 0.790 |
|  | Persistent AF | 1.99 (1.45, 2.73) | <0.001 |  |
|  | **CHA_2_DS_2_-VASc** |  |  |  |
|  | Low | 0.89 (1.05, 3.42) | 0.035 | 0.812 |
|  | High | 2.07 (1.63, 2.63) | <0.001 |  |
| AF/AFL/AT | **Age** |  |  |  |
|  | <65 years | 1.66 (1.05, 2.64) | 0.031 | 0.265 |
|  | ≥65 years | 2.37 (1.81, 3.11) | <0.001 |  |
|  | **Sex** |  |  |  |
|  | Female | 2.16 (1.49, 3.14) | <0.001 | 0.937 |
|  | Male | 2.21 (1.64, 2.99) | <0.001 |  |
|  | **AF type** |  |  |  |
|  | Paroxysmal AF | 2.28 (1.64, 3.16) | <0.001 | 0.795 |
|  | Persistent AF | 2.19 (1.57, 3.06) | <0.001 |  |
|  | **CHA_2_DS_2_-VASc** |  |  |  |
|  | Low | 2.03 (1.11, 3.74) | 0.022 | 0.807 |
|  | High | 2.26 (1.76, 2.90) | <0.001 |  |

High CHA_2_DS₂-VASc risk was defined as a score ≥2. The group which was defined as having no ERAT after 90-day blanking period of catheter ablatio was used as the reference group for all comparisons.

Abbreviations: aHR, adjusted hazard ratio; AF, atrial fibrillation; AFL, atrial flutter; AT, atrial tachycardia; CI, confidence interval.


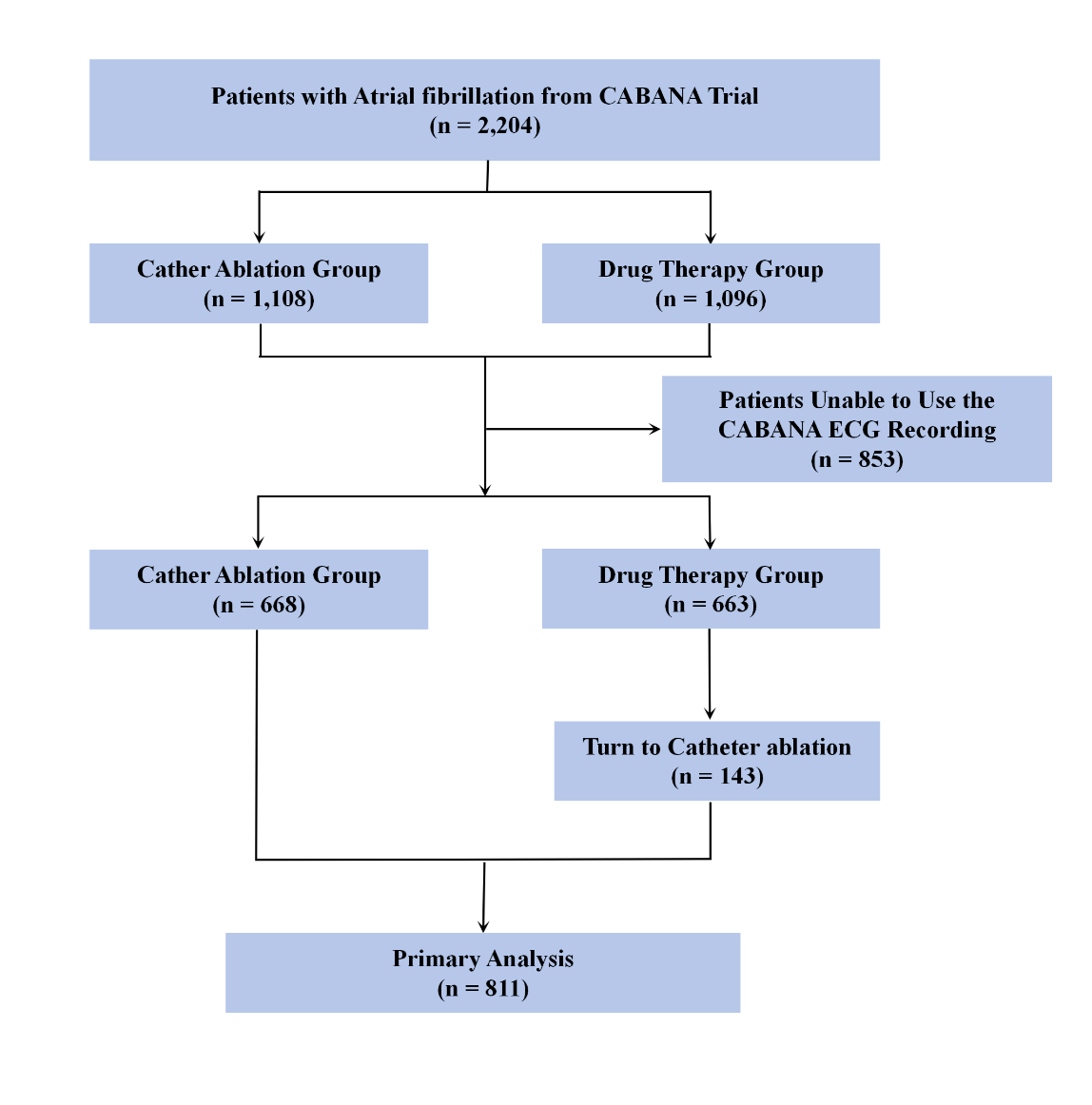


Supplementary Figure S1. The flowchart of this study.

Abbreviations: CABANA, Catheter Ablation vs. Antiarrhythmic Drug Therapy for Atrial Fibrillation; ECG, electrocardiography.


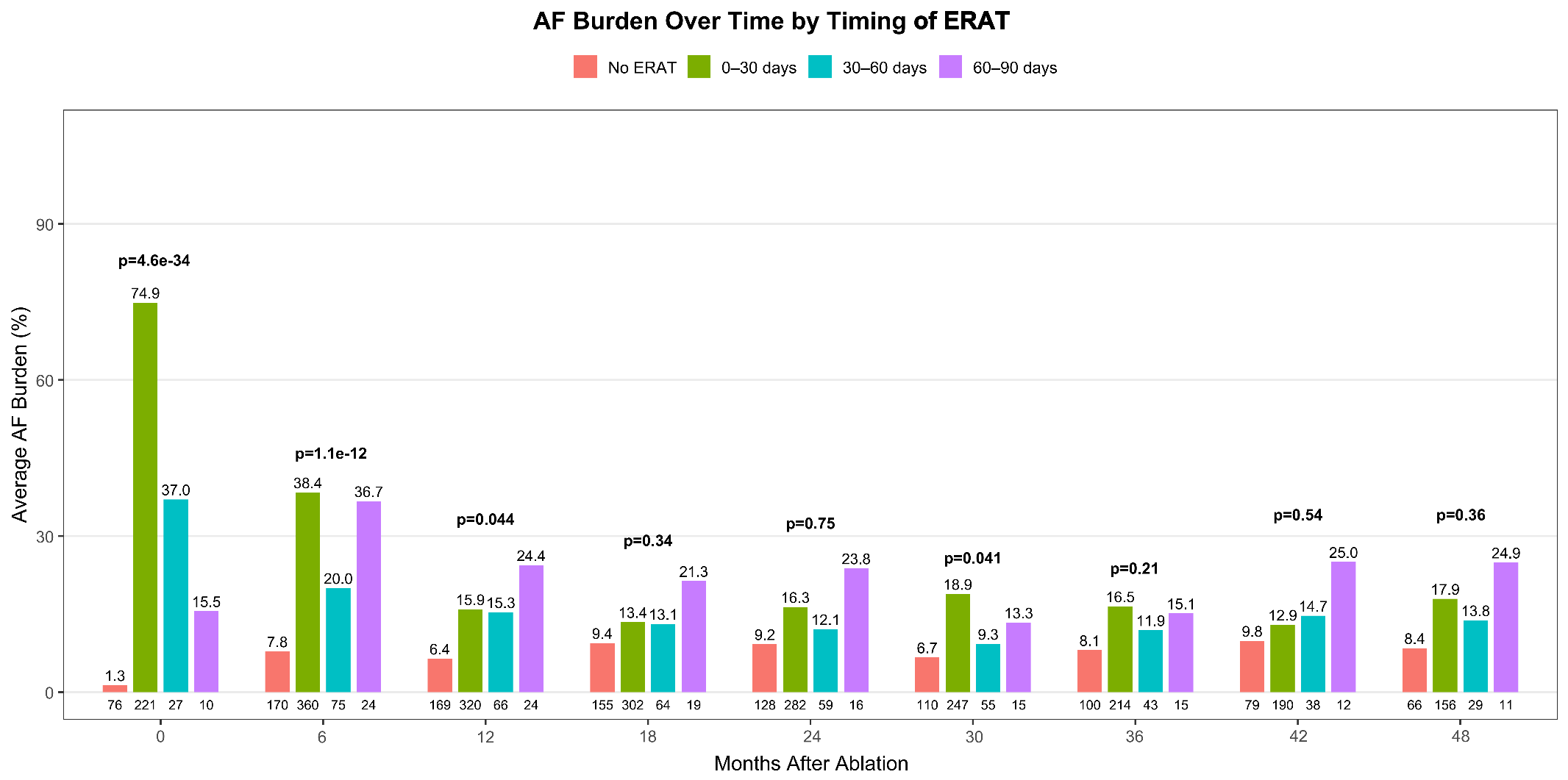


Supplementary Figure S2. The atrial fibrillation burden of participant accepted catheter ablation according to timing of ERAT during the 48-months follow-up.

Abbreviations: AF, atrial fibrillation; ERAT, early recurrence of atrial tachyarrhythmia


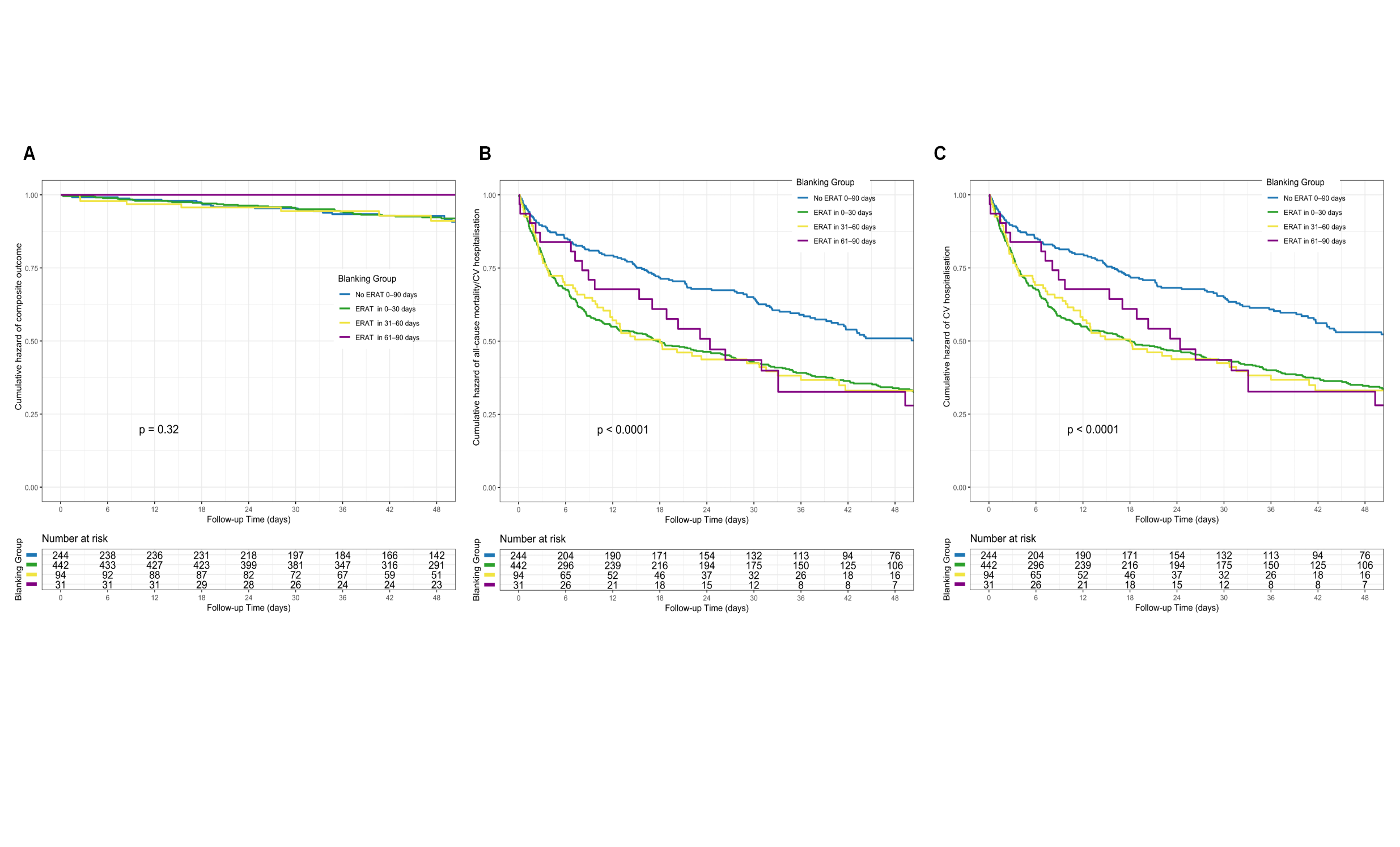
Supplementary Figure S3. Cumulative clinical events curve in AF patients with different timing of ERAT.

A. The cumulative hazard of composite events; B. The cumulative hazard of all-cause mortality or cardiovascular hospitalization; C. The cumulative hazard of cardiovascular hospitalization.

Composite events was a composite endpoint including all-cause mortality, disabling stroke, cardiac arrest or serious bleeding events.

Abbreviations: CV hospitalization, cardiovascular hospitalization; ERAT, early recurrence of atrial tachyarrhythmia.


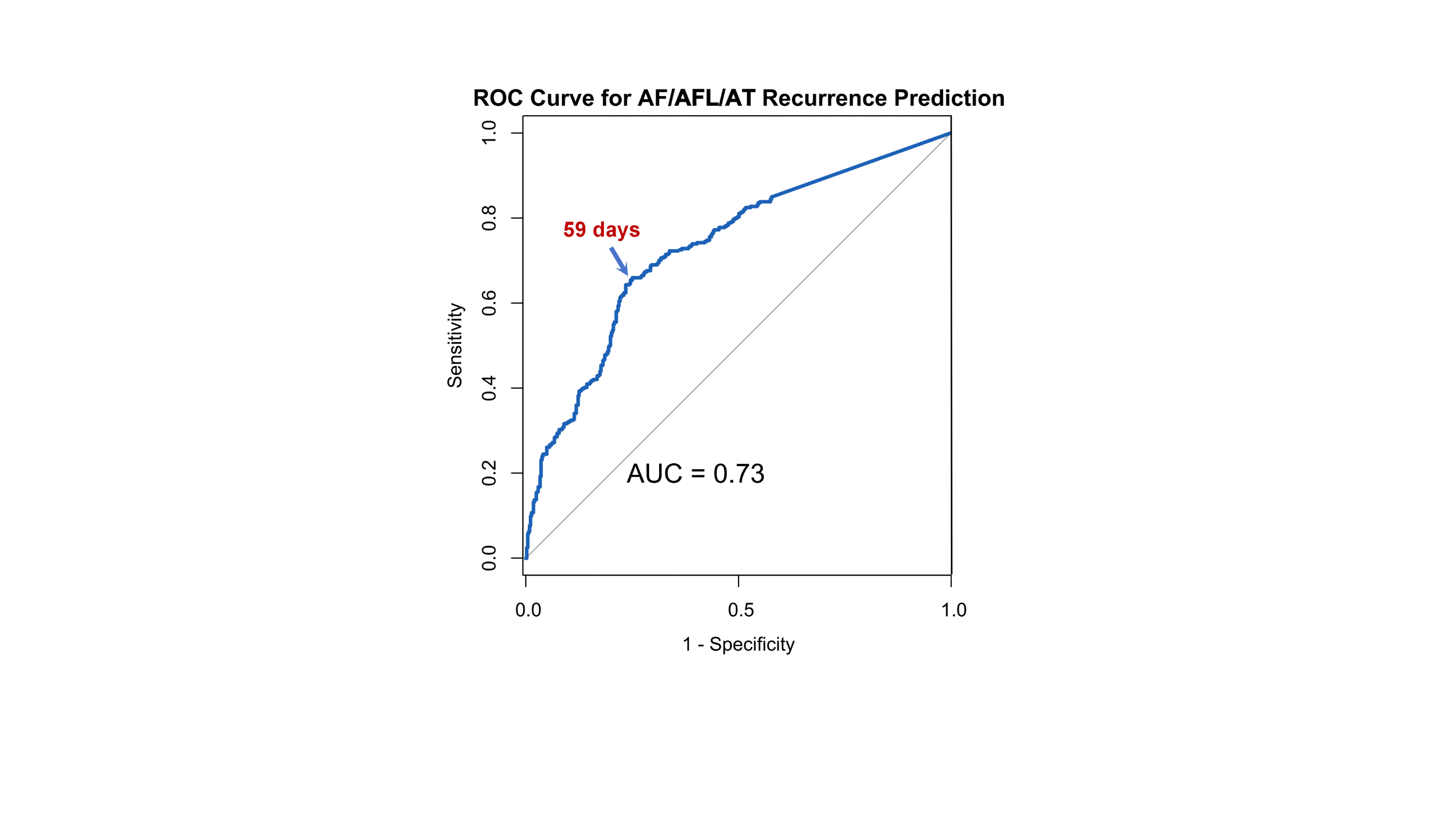


Supplementary Figure S4. Receiver operating characteristic (ROC) curve for optimal duration of blanking period (AF/AFL/AT).

Abbreviations: AF, atrial fibrillation; AFL, atrial flutter; AT, atrial tachycardia; AUC, area under the curve; ROC, receiver operating characteristic.

Sensitivity: 0.659; Specificity: 0.749
